# Supplementary material for: Carbon Ion Irradiation Downregulates Notch Signaling in Glioma Cell Lines, Impacting Cell Migration and Spheroid Formation
Source: Cells. 2022 Oct 24;11(21):3354. doi: 10.3390/cells11213354 (PMC9655483; doi:10.3390/cells11213354)
Supplement: Supplementary file 1 [file cells-11-03354-s001.zip › cells-1895497-supplementary.pdf]

Carbon Ion Irradiation downregulates Notch signaling in glioma cell lines, impacting cell migration and spheroid formation.

Supplementary Figures

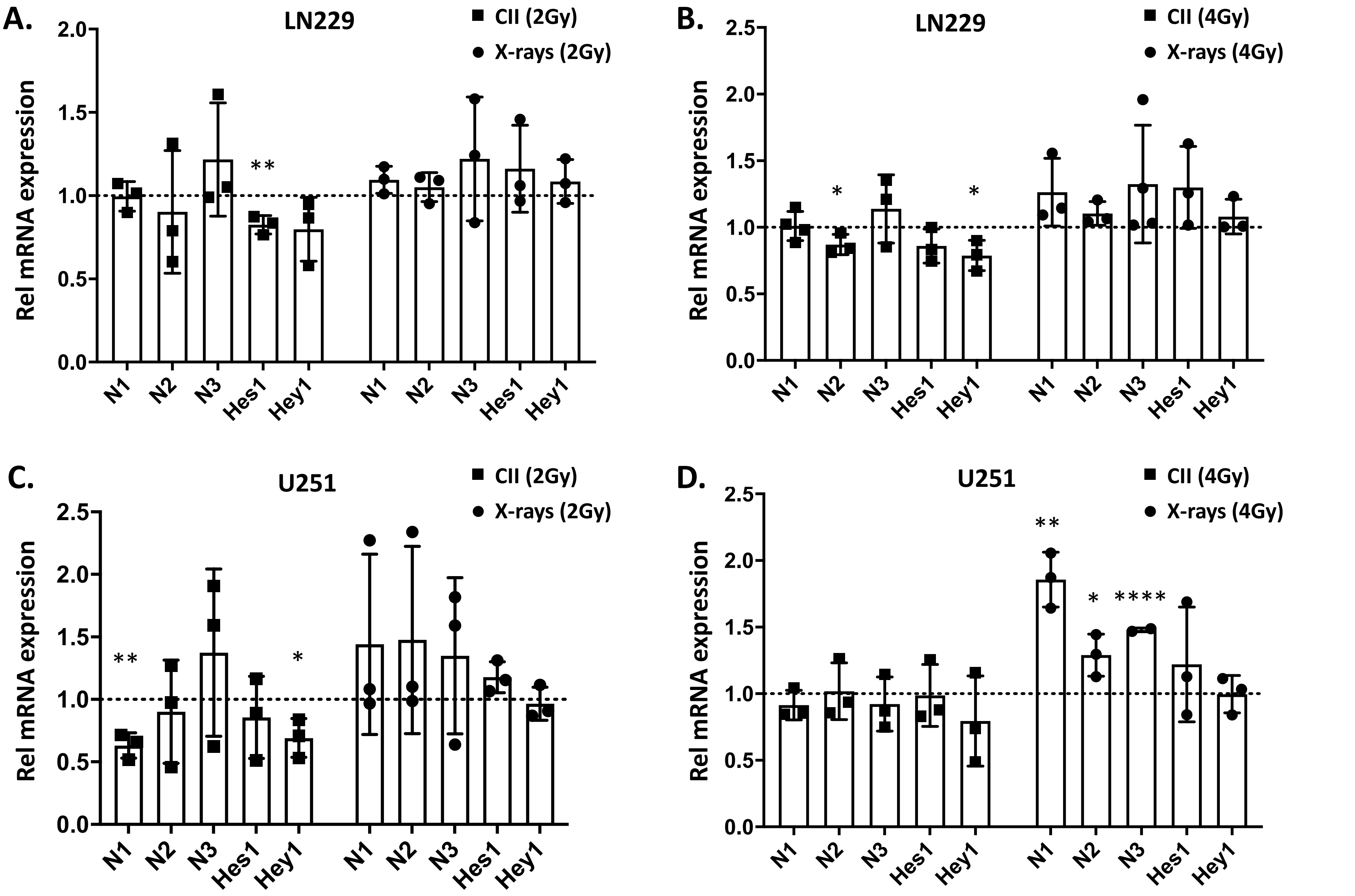

Figure S1. Differential regulation of Notch signaling genes by CII and X-Ray at 6h in human glioma cell lines: The mRNA expression of Notch Receptors (relative to un-irradiated sample): *Notch1* (N1), *Notch2* (N2) and *Notch3* (N3); Notch Target genes: *Hes1* and *Hey1*, observed at 6 h after irradiation were analyzed by qRT-PCR in LN229 and U251 monolayer cell culture irradiated with either (A)(C) 2 Gy X-ray or Carbon Ion irradiation or (B)(D) 4 Gy X-ray or Carbon Ion irradiation. Each symbol represents an independent experiment done in triplicates. Data are presented as Mean ±SD. p values were determined by an unpaired two-tailed Student's t-test, *$p < 0.05$, **$p < 0.01$, ****$p < 0.0001$.

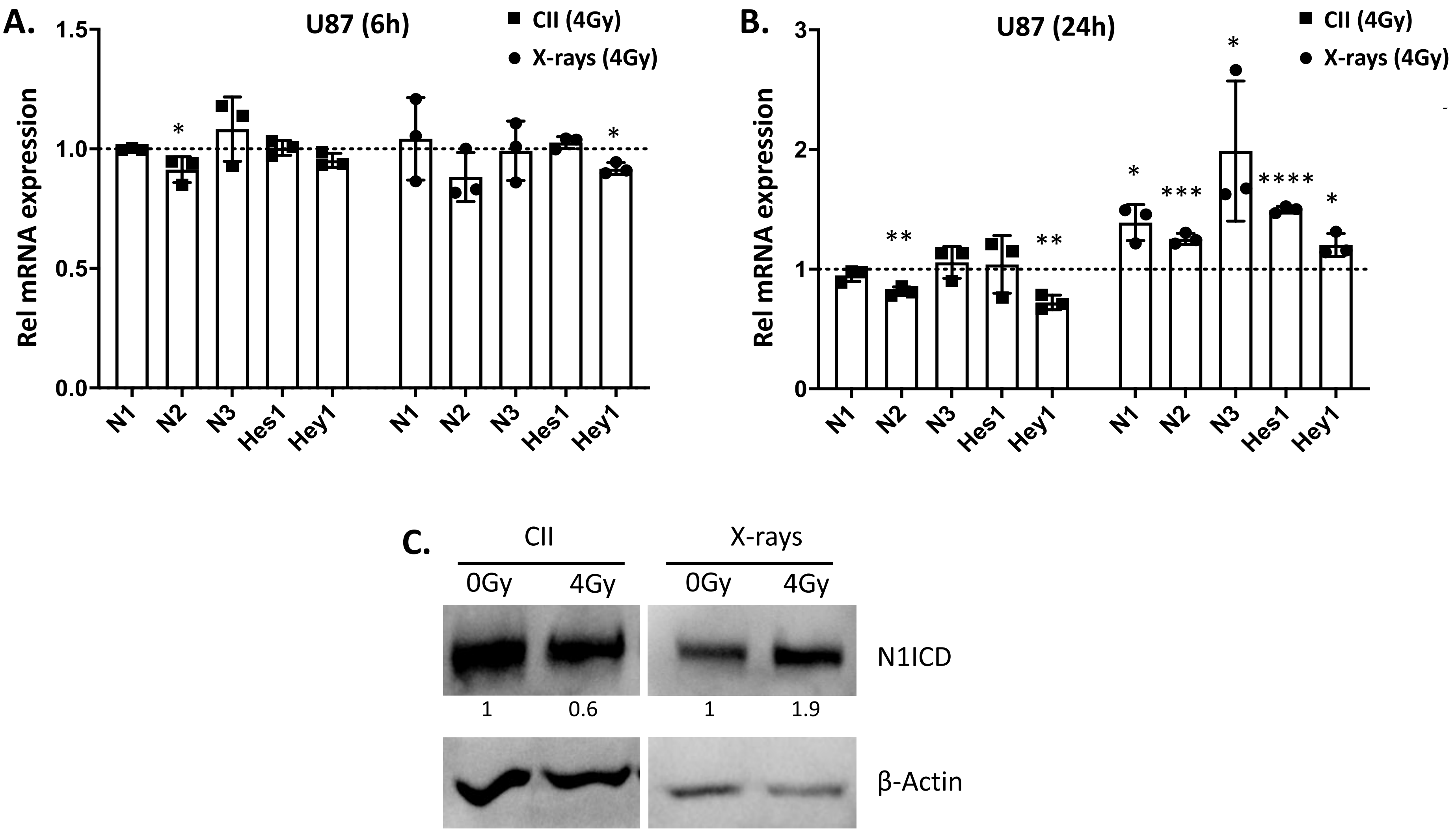

Figure S2: Differential regulation of Notch signaling genes and N1ICD by CII and X-Ray in U87 cell lines: The mRNA expression of Notch Receptors (relative to un-irradiated sample): *Notch1* (N1), *Notch2* (N2) and *Notch3* (N3); Notch Target genes: *Hes1* and *Hey1*, observed at (A) 6 h or (B) 24h after irradiation were analyzed by qRT-PCR in U87 monolayer cell culture irradiated with either 4Gy X-ray or 4Gy Carbon Ion irradiation. (C) Western blot analysis of NOTCH1 and β-ACTIN as a loading control in U87 cells irradiated with either CII or X-rays. Each symbol represents an independent experiment done in triplicates. Data are presented as Mean ±SD. *P* values were determined by an unpaired two-tailed Student's t-test, *$p < 0.05$, **$p < 0.01$, ***$p < 0.001$, ****$p < 0.0001$.

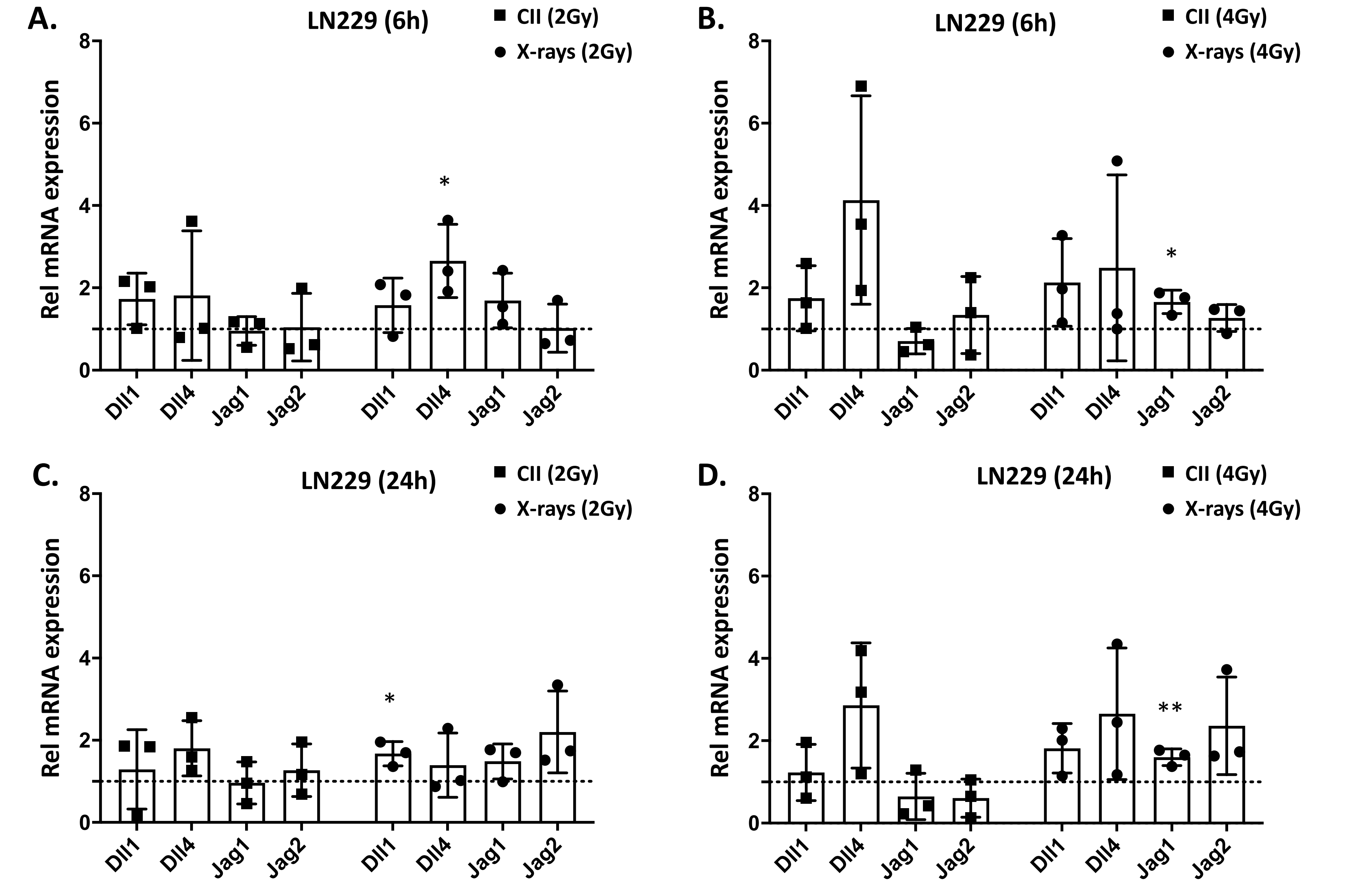

Figure S3. Effect of CII and X-rays on mRNA expression of Notch ligands genes at 6h and 24h in LN229 cell line: The mRNA expression of Notch ligands (relative to un-irradiated sample): *Delta like 1 (Dll1)*, *Delta like 4 (Dll4)*, *Jagged1 (Jag1)* and *Jagged2 (Jag2)* were analyzed by qRT-PCR in LN229 monolayer cell culture irradiated with either (A)(C) 2 Gy X-ray or Carbon Ion irradiation at 6h and 24h or (B)(D) 4 Gy X-ray or Carbon Ion irradiation at 6h and 24h. Each symbol represents an independent experiment done in duplicates. Data are presented as Mean ±SD. *p* values were determined by an unpaired two-tailed Student's t-test, *$p < 0.05$, **$p < 0.01$.

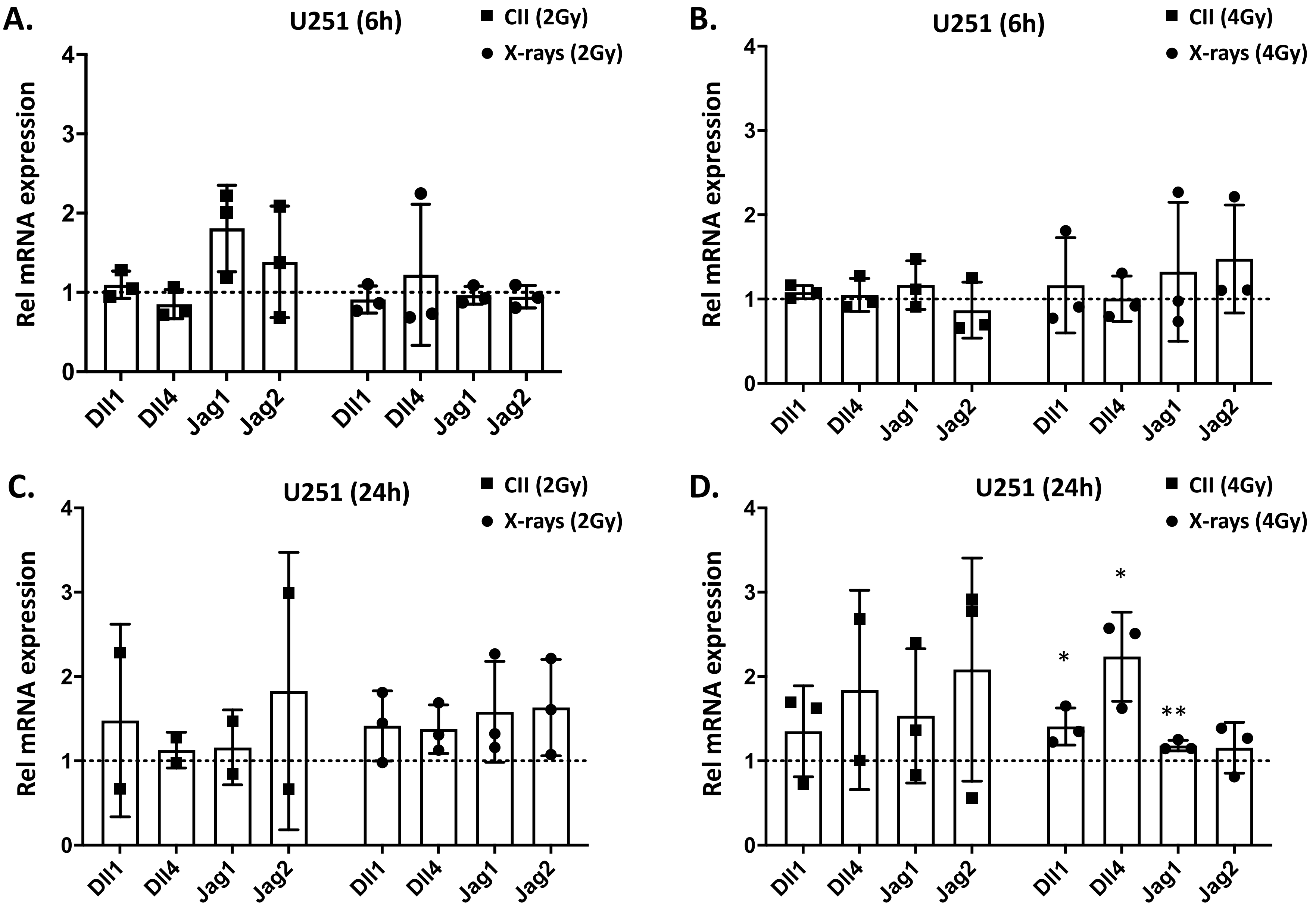

Figure S4. Effect of CII and X-rays on mRNA expression of Notch ligands genes at 6h and 24h in U251 cell line: The mRNA expression of Notch ligands (relative to un-irradiated sample): *Delta like 1 (Dll1)*, *Delta like 4 (Dll4)*, *Jagged1 (Jag1)* and *Jagged2 (Jag2)* were analyzed by qRT-PCR in U251 monolayer cell culture irradiated with either (A)(C) 2 Gy X-ray or Carbon Ion irradiation at 6h and 24h or (B)(D) 4 Gy X-ray or Carbon Ion irradiation at 6h and 24h. Each symbol represents an independent experiment done in duplicates. Data are presented as Mean ±SD. *p* values were determined by an unpaired two-tailed Student's t-test, *$p < 0.05$, **$p < 0.01$.

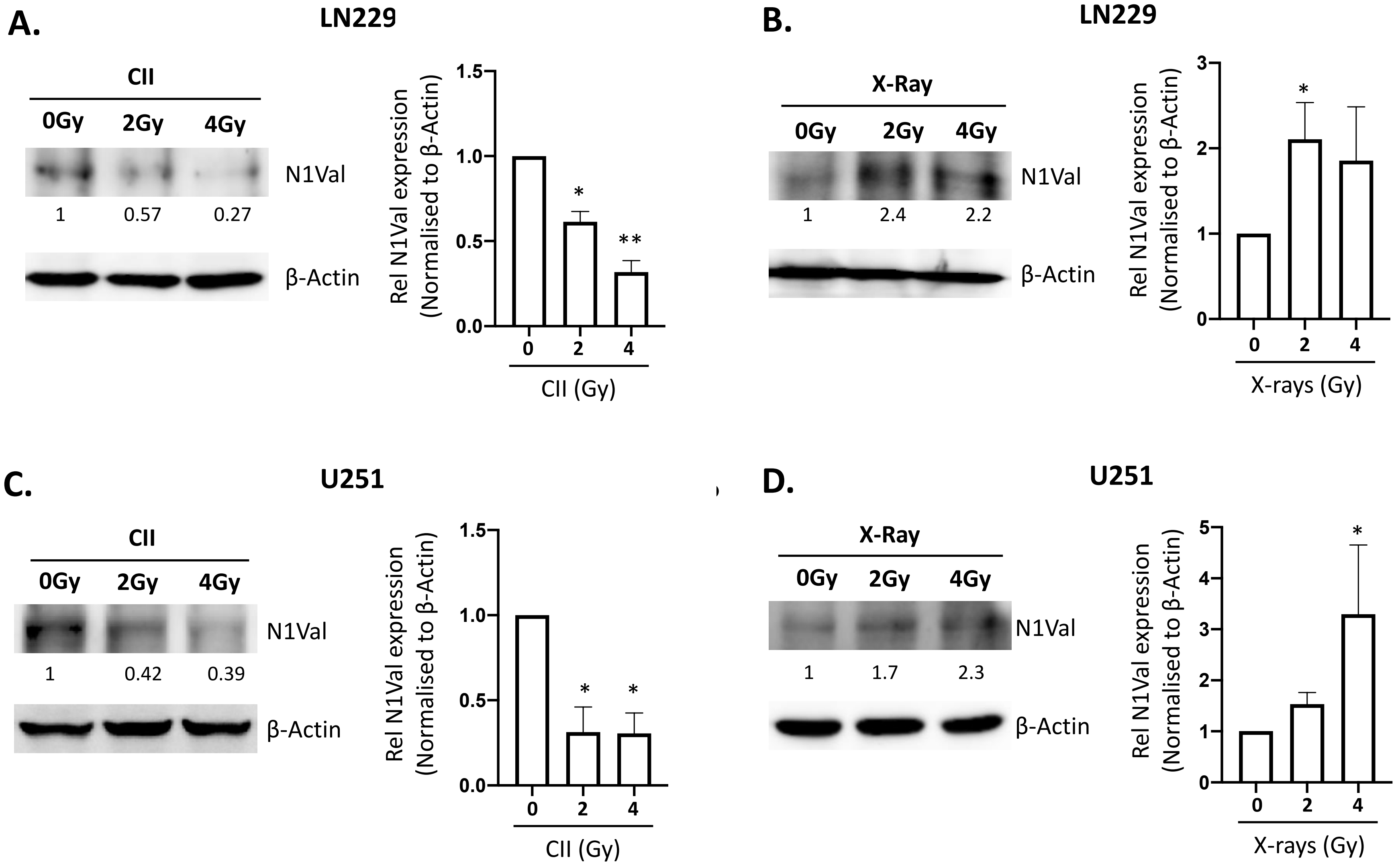

Figure S5: CII reduces, while X-Ray enhances Notch1 activation: Western blot analysis of NOTCH1-Val (Val-1744) and β-actin as a loading control in (A) LN229 irradiated with CII, (right) densitometry analysis of protein level in panel A; values are average of two independent experiments. (B)) LN229 irradiated with X-Ray, (right) densitometry analysis of protein level in panel B, values are average of two independent experiments; (C)) U251 irradiated with CII, (right) densitometry analysis of protein level in panel C, values are average of two independent experiments.; (D) U251 irradiated with X-Ray, (right) densitometry analysis of protein level in panel D, values are average of two independent experiments. Data are presented as mean ±SD. Statistical analysis was performed by ANOVA with Bonferroni post-hoc test. *p*-value (*$p < 0.05$, **$p < 0.01$).

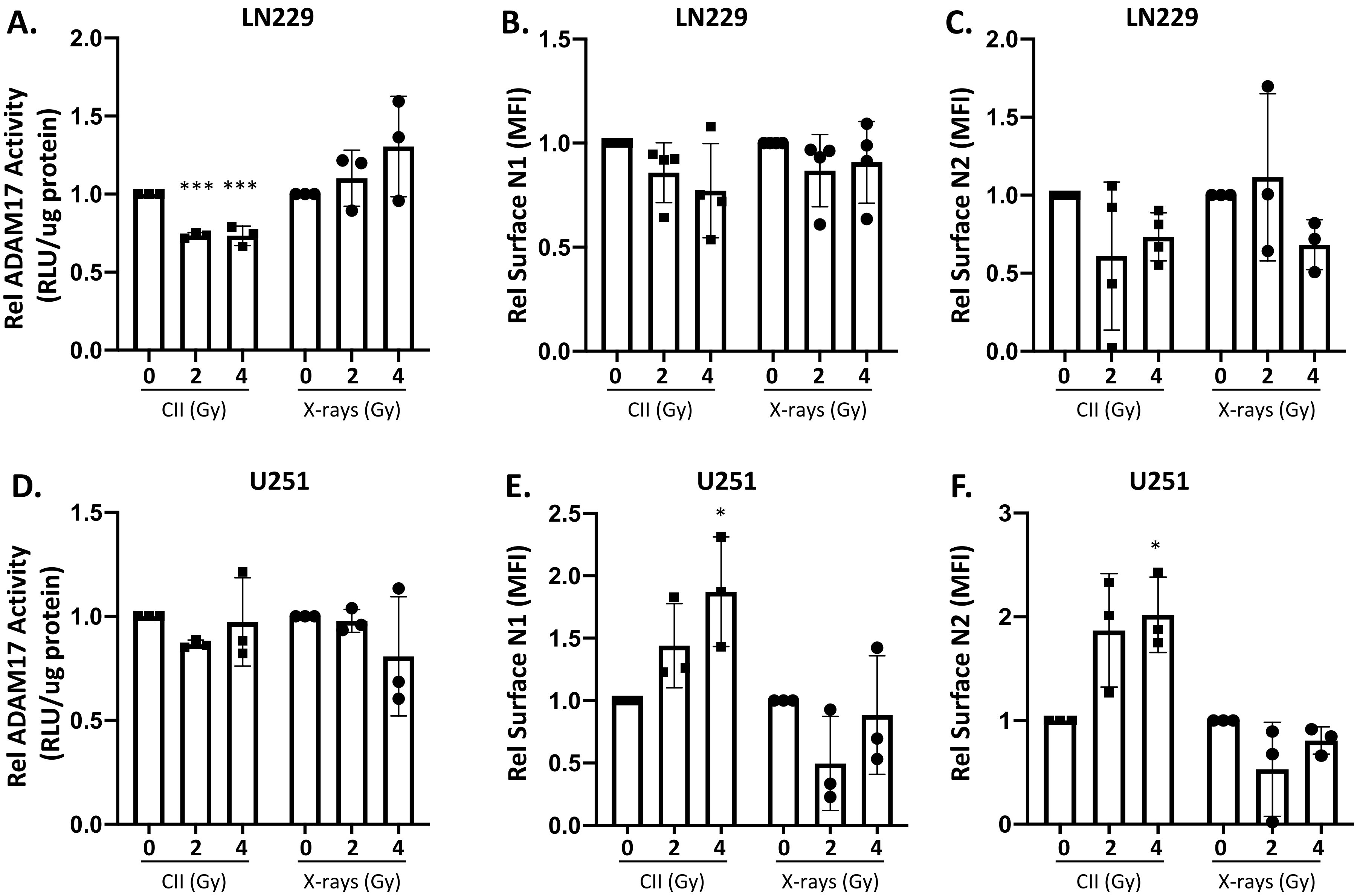

Figure S6. Differential regulation of ADAM17 activity and cell surface Notch receptors by CII and X-rays in human glioma cell lines at 6h: Relative ADAM17 activity after irradiation with X-Ray and CII in (A) LN229 and (D) U251 cells. Relative MFI of cell surface Notch1 in (B) LN229 and (E) U251, and Notch2 in (C) LN229 and (F) U251,as determined by flow cytometry. Each symbol represents an independent experiment. Data are presented as Mean ±SD. Statistical analysis was performed by ANOVA with Bonferroni post-hoc test. *p*-value (*$p < 0.05$, ***$p < 0.001$).

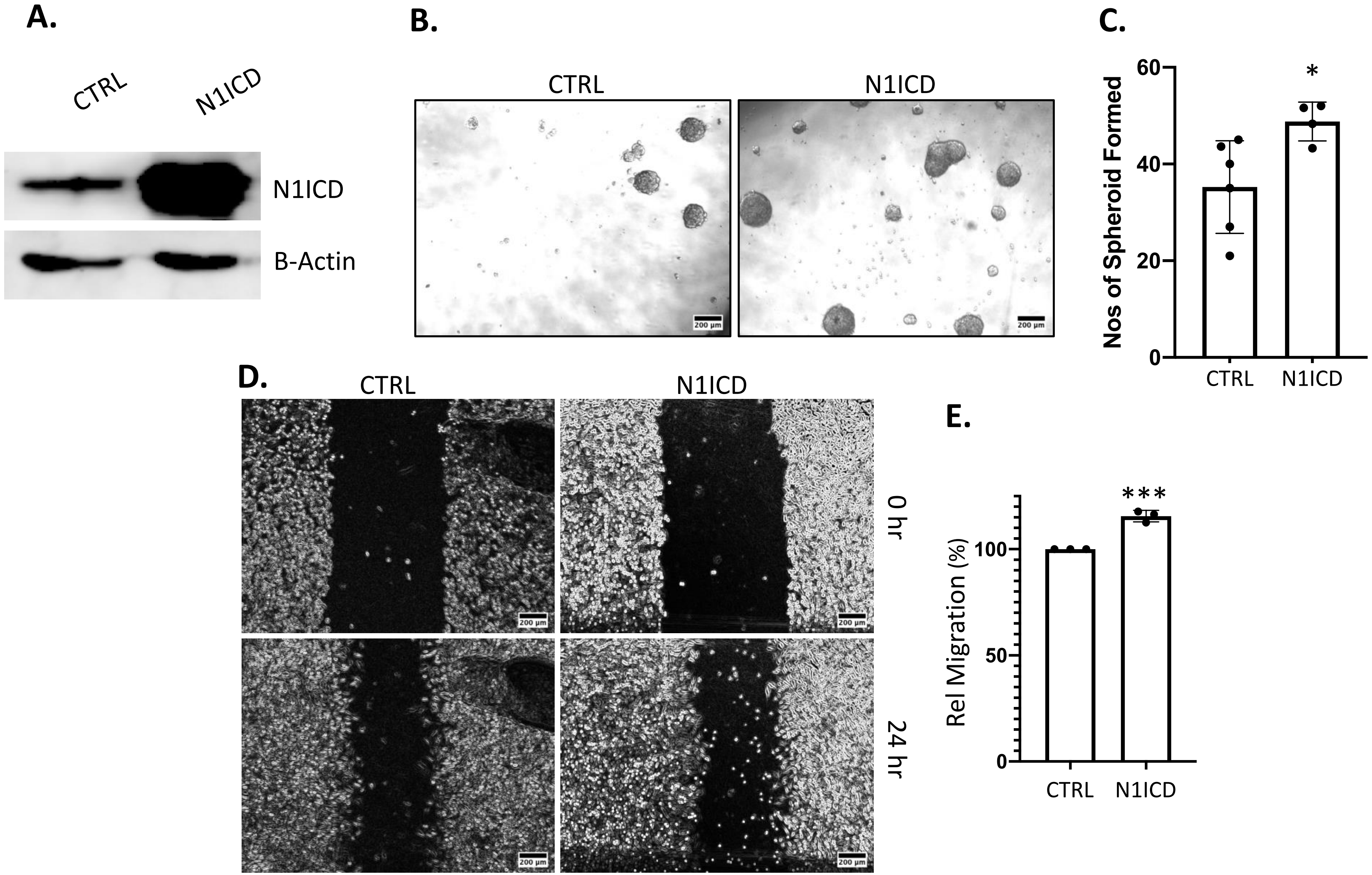

Figure S7. Over-expression of N1ICD in LN229 increases spheroid formation and migration in a human glioma cell line LN229: (A) Expression of Notch1ICD in Control and N1ICD overexpressing LN229 cells as determined by western blot. (B) Representative microscopic image of the spheroid formed at day 10-14 (scale bar in black = 200µm). (C) Quantification of spheroid number derived from (B). Each experiment was done in triplicate. (D) Representative images of the migration of Control or N1ICD overexpressing LN229 cells, as analyzed by scratch assay (scale bar in black = 200µm). (E) Bar graph showing relative migration in percentage as derived from (D). Each symbol represents an independent experiment done in duplicates. Data are presented as mean ±SD. *p* values were determined by an unpaired two-tailed Student's t-test, *$p < 0.05$, ***$p < 0.001$.

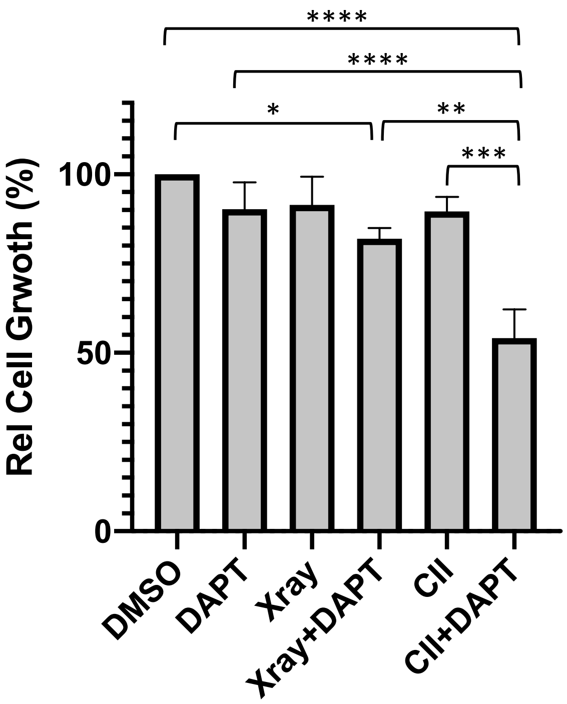

Figure S8. Effect of DAPT on Xray or CII induced growth inhibition observed at 48 hr : (A) Relative cell growth at 48 hours after treatment with DAPT (10uM) or Xray (2Gy) or CII (2Gy) alone or in combination of DAPT(10uM) with CII(2Gy) or X-rays (2Gy). Values are average of two independent experiments. Data are presented as mean ±SD. Statistical analysis was performed by ANOVA with Bonferroni post-hoc test. *p*-value (* $p < 0.05$, ** $p < 0.01$, *** $p < 0.001$, **** $p < 0.0001$).
